# Effects of Sit-to-stand Exercise Instruction and Goal-setting on Reducing Sitting Time in Chronic Stroke Survivors: A Randomized Crossover Trial

**DOI:** 10.1298/ptr.25-E10362

**Published:** 2026-06-10

**Authors:** Mitsutaka SHIBUYA, Kazuhiro HARADA, Yohsuke YAMASHITA, Katsuki TAKAOKA, Keiji IKEWAKI, Kenichi HIRASHIMA, Haruo UGUISU

**Affiliations:** 1 Department of Physical Therapy, Faculty of Health and Welfare, Tokushima Bunri University, Japan; 2 Graduate School of Health Science Studies, Kibi International University, Japan; 3 Hashimoto Rehabilitation Clinic, Japan; 4 Long-Life Care Section, Health and Welfare Division, Naruto City Hall, Japan; 5 Department of Rehabilitation Technology, Tokushima Prefectural Central Hospital, Japan

**Keywords:** Chronic stroke, Sedentary behavior, Sit-to-stand, Crossover trial

## Abstract

**Objectives:**

This study compared the effects of sit-to-stand exercise instruction (STS instruction) and participant-led goal-setting on sedentary behavior in chronic stroke survivors with reduced walking ability. We hypothesized that STS instruction would reduce prolonged sitting bouts more effectively than goal-setting.

**Methods:**

Twelve chronic stroke survivors completed a randomized crossover trial consisting of two 2-week interventions (STS instruction and participant-led goal-setting) separated by a 1-month washout. Physical activity time was measured using a thigh-worn activPAL4 accelerometer. The primary outcomes were the number and total duration of prolonged sitting bouts ≥30 min. Secondary outcomes included total sitting/lying time, standing/walking time, sit-to-stand transitions, and prolonged sitting bouts ≥60 min.

**Results:**

A significant intervention × time interaction was observed for prolonged sitting bouts ≥30 min (p = 0.049), with different pre–post patterns between intervention phases (STS: 8.4–7.0/day; goal-setting: 8.1–9.3/day). The interaction for the total duration of ≥30-min bouts was not statistically significant (p = 0.156). No significant intervention × time interactions were observed for total sedentary time, standing/walking time, or for the number or total duration of sitting bouts ≥60 min.

**Conclusions:**

STS instruction may contribute to modifying sitting patterns in chronic stroke survivors with limited walking ability, even without changes in total sedentary or upright time. These preliminary findings suggest that a simple, task-oriented approach may help modify sedentary patterns, although clear superiority over participant-led goal-setting was not demonstrated. Larger and longer-term trials are needed.

## Introduction

Stroke survivors in the chronic phase exhibit markedly low physical activity and a sedentary lifestyle. They walk, climb stairs, and change posture at about half the frequency of healthier older adults^1,2)^, and they spend 69%–87% of waking hours sedentary^3–6)^, often in prolonged, uninterrupted bouts^5)^. These patterns persist for years and contribute to functional decline and the risk of long-term stroke recurrence^7)^. Although the World Health Organization recommends reducing prolonged sitting^8)^, increasing ambulatory activity is challenging for people with substantial mobility limitations. Walking speed strongly influences daily steps^9)^, and individuals walking <0.8 m/s show markedly low activity and high sedentary time^10)^. Thus, interrupting sedentary time—rather than increasing steps—may be a more feasible behavioral target for this population^11)^. Interventions intended to modify physical activity or sedentary behavior after stroke have used exercise-based, behavioral, and educational strategies^12)^. Exercise-based approaches focusing on frequent standing showed short-term feasibility in subacute stroke^13,14)^, whereas repeated encouragement or counseling produced mixed results in chronic survivors^15)^. Participant-led goal-setting may enhance motivation and support behavioral change^16–19)^, yet its effectiveness in individuals with greater disability or reduced walking speed remains unclear. Chronic stroke survivors with limited gait ability may be unable to increase overall activity yet remain capable of modifying sedentary patterns—particularly prolonged sitting—through achievable, task-oriented movements. Practicing sit-to-stand movements directly increases postural transitions and may interrupt sedentary accumulation^13,14)^. Therefore, sit-to-stand exercise instruction (STS instruction) may be expected to induce more immediate postural changes than the more indirect behavioral approach of participant-led goal-setting. Accordingly, we hypothesized that STS instruction would reduce prolonged sitting bouts more than participant-led goal-setting. To date, no study has directly compared a task-oriented movement strategy with a participant-led behavioral strategy in chronic stroke survivors with reduced walking ability. This study compared their short-term effects on sedentary behavior patterns using thigh-worn accelerometry.

## Methods

### Study design

A randomized crossover design was used to compare STS instruction with participant-led goal-setting. The trial was registered with UMIN (UMIN000043762). The allocation, crossover sequence, and analysis are shown in Figure 1, while a CONSORT flow diagram illustrating screening is provided in Figure 2.

**Fig. 1.**
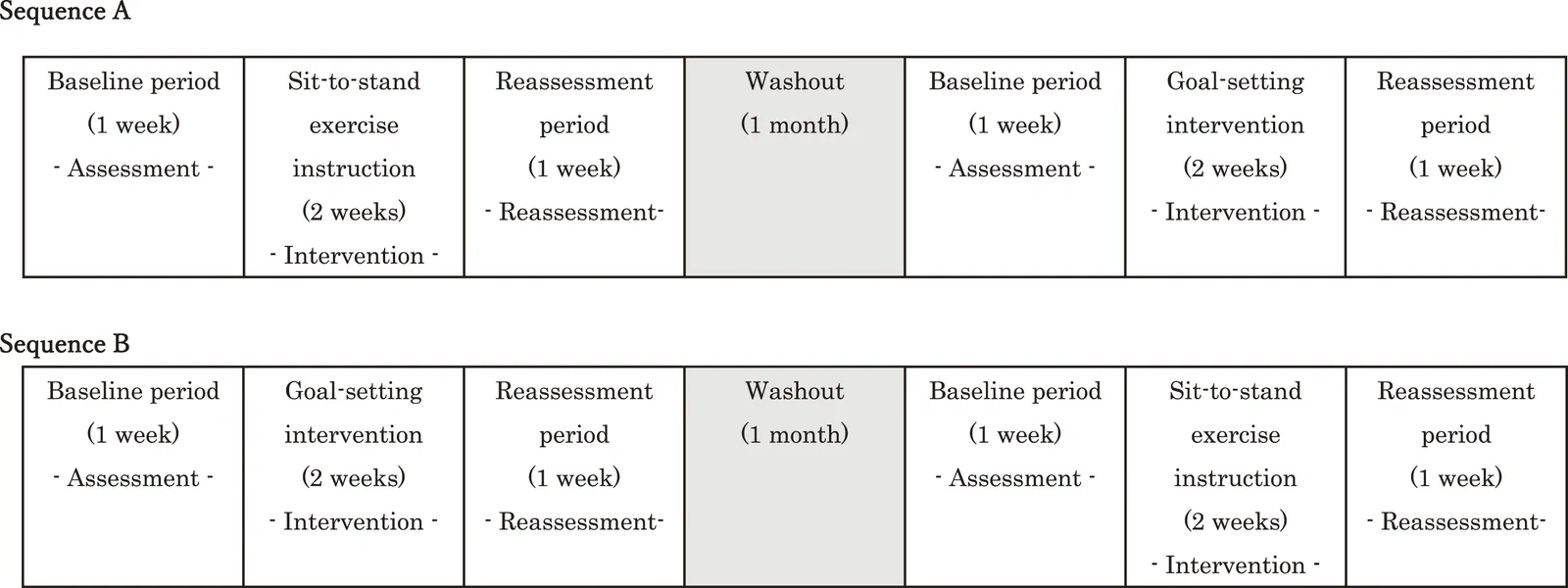
Study design Crossover trial showing 2 intervention sequences: Sequence A, STS instruction → goal-setting sequence; Sequence B, goal-setting → STS instruction sequence. STS, sit-to-stand exercise

**Fig. 2.**
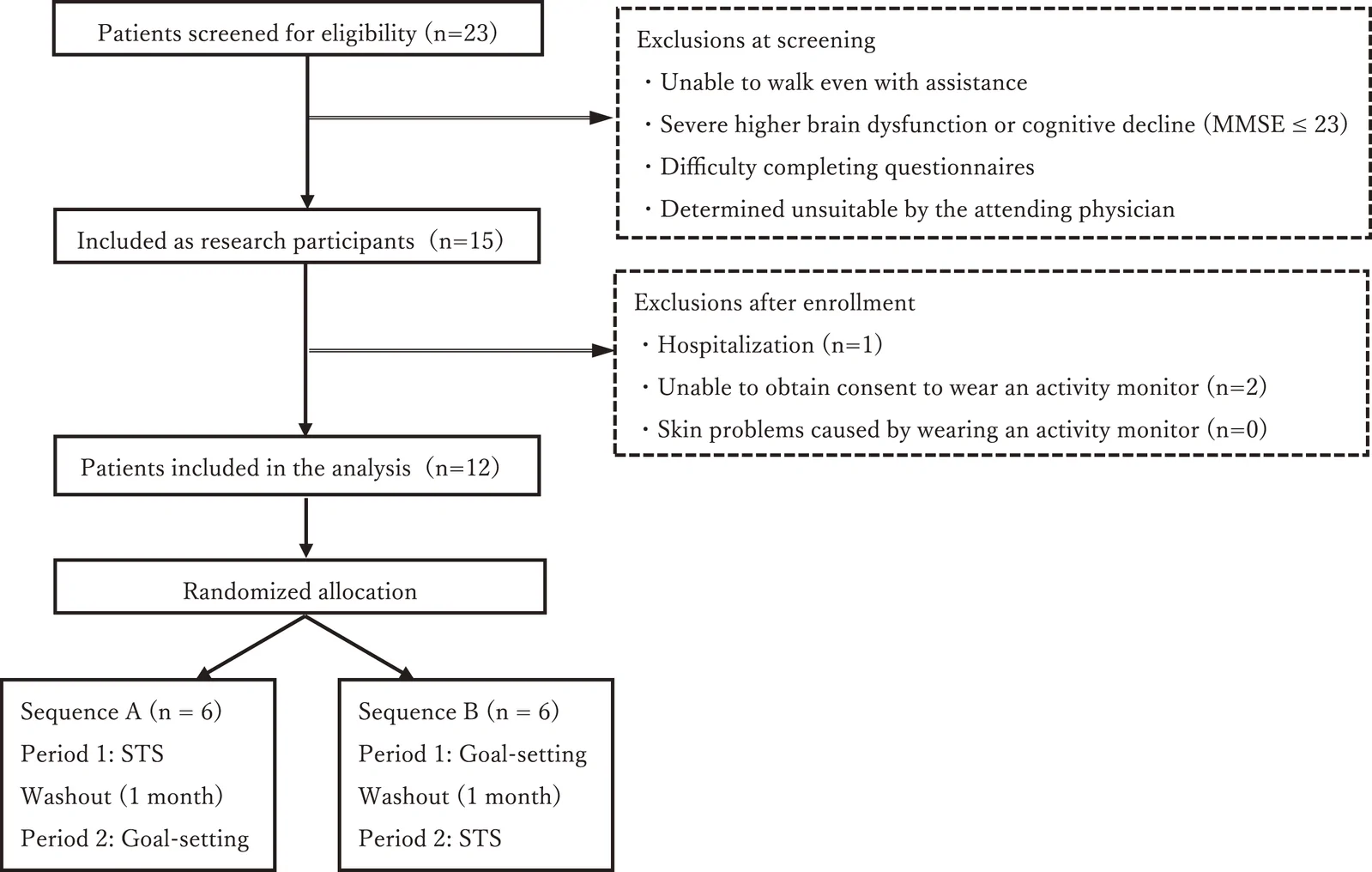
Participant flow and allocation to crossover sequences. MMSE, Mini-Mental State Examination; STS, sit-to-stand exercise

### Participants

Stroke survivors attending the Hashimoto Rehabilitation Clinic between April 2021 and March 2022 were screened and provided written informed consent.

### Inclusion criteria

- Stroke onset >6 months prior
- Indoor ambulation with supervision/light assistance (Functional Ambulation Categories [FAC] 2–4)
- Independent sit-to-stand ability
- Capacity to wear activPAL (PAL Technologies, Glasgow, UK) continuously

### Exclusion criteria

- Higher brain dysfunction impairing comprehension (Mini-Mental State Examination [MMSE] score ≤23, attention/memory deficits, aphasia, unilateral neglect, or clinician-judged unsuitability)
- Severe walking limitation (FAC 0–1)

### Sample size

This exploratory study did not include an a priori sample size calculation. A post-hoc estimation based on the observed interaction effect indicated that 18 participants would be required to achieve 80% power; therefore, the final sample (n = 12) was smaller than the estimated requirement for detecting small effects.

### Measurements

#### Participant characteristics

Baseline characteristics included demographics, body mass index (BMI), MMSE, National Institutes of Health Stroke Scale (NIHSS), Stroke Impairment Assessment Set (SIAS), and FAC. Psychological measures included the Yaruki score^20)^, Vitality Index^21)^, 15-item Geriatric Depression Scale (GDS)^22)^, and the Transtheoretical Model^23)^.

#### Motor function

Baseline motor function included non-paretic grip strength/body-weight ratio, walking speed, 6-minute walk test (6MWT), and the Ability for Basic Movement Scale (ABMS) II. Motor outcomes were not analyzed longitudinally.

#### Physical activity measurement

Physical activity was measured using a thigh-mounted triaxial accelerometer (activPAL4; PAL Technologies; 20-Hz sampling rate). The device was attached to the anterior non-paretic thigh with a waterproof dressing and worn continuously for 5 days per phase, including sleep, producing 24-h monitoring data. Valid data required ≥1440 min/day (24 h) for at least 3 days. To reflect home behavior, device attachment and removal were performed at the rehabilitation center on days participants attended day-rehabilitation services. Outcomes included total sitting/lying time, standing/walking time, sit-to-stand transitions, and sitting bouts ≥30 and ≥60 min.

### Interventions

#### STS instruction

Participants were instructed to perform sit-to-stand movements at home every day at a comfortable pace from a standard chair, using hand support or a handrail as needed. They performed 30 repetitions per day (10 repetitions each in the morning, afternoon, and evening), taking short rests between sets as required. Adherence was recorded using daily checklists.

#### Goal-setting

A participant-led, non-directive goal-setting approach was used. Participants selected feasible daily strategies (e.g., standing during TV commercials, brief post-meal walking) through therapist-guided, open-ended questions. Participants self-monitored their goal attainment, and no feedback or reminders were provided during the intervention period to avoid influencing their autonomous decision-making.

#### Usual rehabilitation

Routine rehabilitation continued without modification.

### Ethics

The study was approved by the Ethical Review Committee of Kibi International University (No. 20-53). Written informed consent was obtained from all participants.

### Statistical analysis

Analyses were performed using R version 4.3.3 (R Foundation for Statistical Computing, Vienna, Austria) with the R Commander package. We fitted mixed-effects models for repeated measures (MMRM) including sequence, period, intervention, time (pre/post), and the intervention × time interaction as fixed effects, with the participant as a random intercept. Model-based estimated marginal means were used to derive within-intervention changes (Δ) and the between-intervention difference in change (ΔΔ; i.e., interaction contrast) with 95% confidence intervals (CIs). Models were fitted using restricted maximum likelihood, and p <0.05 was considered statistically significant.

## Results

### Participant characteristics

Fifteen participants were enrolled, and 12 completed the trial. Baseline characteristics are shown in Table 1. Cognitive function was preserved, mean gait independence was FAC 3, and stroke severity was mild to moderate. Psychological assessments indicated moderate motivation and mild depressive symptoms. Baseline GDS-15 scores were similar between sequences (Sequence A: 5.2 ± 3.3; Sequence B: 5.7 ± 4.0; Table 1). Given the small sample size, baseline comparisons were interpreted descriptively; standardized mean differences (SMDs) are provided to indicate the magnitude of potential imbalance between sequences (Table 1).

**Table 1.** Baseline characteristics of participants by sequence

Outcome	Total (n = 12)	Sequence A (n = 6)	Sequence B (n = 6)	p Value	SMD (A−B)
Age (years)	73.1 ± 4.9	73.5 ± 1.2	72.7 ± 7.1	0.79	0.15
Sex (male/female)	11/1	5/1	6/0	1.00	–
BMI (kg/m^2^)	22.4 ± 2.2	22.6 ± 2.4	22.2 ± 2.2	0.78	0.15
MMSE (0–30)	28.8 ± 1.5	28.7 ± 2.0	28.8 ± 1.3	0.87	−0.09
Time since onset (years)	5.3 ± 4.1	3.8 ± 4.7	6.7 ± 3.3	0.25	−0.65
FAC	2.8 ± 1.1	3.2 ± 1.2	2.5 ± 1.0	0.32	0.55
NIHSS (points)	4.2 ± 3.1	4.3 ± 3.9	4.2 ± 2.5	0.93	0.05
SIAS (points)	46.2 ± 12.8	47.2 ± 14.6	45.3 ± 11.9	0.82	0.13
Yaruki score	16.3 ± 8.7	16.3 ± 9.2	16.2 ± 9.0	0.98	0.02
Vitality Index	8.9 ± 1.1	8.7 ± 1.2	9.2 ± 1.0	0.45	−0.42
GDS-15 (points)	5.4 ± 3.5	5.2 ± 3.3	5.7 ± 4.0	0.82	−0.13
Transtheoretical model stage	2.8 ± 1.2	3.2 ± 1.5	2.3 ± 0.8	0.26	0.65
Non-paretic grip strength/ body-weight ratio	0.47 ± 0.13	0.45 ± 0.12	0.49 ± 0.14	0.44	−0.29
Comfortable walking speed (m/s)	0.30 ± 0.23	0.37 ± 0.25	0.22 ± 0.17	0.23	0.63
Fastest walking speed (m/s)	0.40 ± 0.32	0.47 ± 0.29	0.32 ± 0.32	0.38	0.42
6MWT (m)	102.5 ± 81.6	129.3 ± 87.7	75.7 ± 70.8	0.24	0.61
ABMS II (5–30)	29.4 ± 0.9	29.7 ± 0.8	29.2 ± 1.0	0.32	0.51

Values are mean ± SD. Welch’s t-test (continuous variables) and Fisher’s exact test (sex) were used for between-sequence comparisons. SMD (Hedges’ g) indicates the magnitude of potential imbalance (positive values indicate higher values in Sequence A). p-Values are descriptive only.

SMD, standardized mean difference; BMI, body mass index; MMSE, Mini-Mental State Examination; FAC, Functional Ambulation Categories; NIHSS, National Institutes of Health Stroke Scale; SIAS, Stroke Impairment Assessment Set; GDS, 15-item Geriatric Depression Scale; Transtheoretical Model stage: 1 = Precontemplation, 2 = Contemplation, 3 = Preparation,4 = Action, 5 = Maintenance; 6MWT, 6-minute walk test; ABMS II, Ability for Basic Movement Scale II; SD, standard deviation

### Physical activity and sedentary behavior (Table 2)

**Table 2.** Effects of STS and goal-setting on daily physical activity and sedentary pattern outcomes: Results from mixed-effects models

Outcome	STS pre-EMM	STS post-EMM	STS Δ (95% CI), p	GS pre-EMM	GS post-EMM	GS Δ (95% CI), p	ΔΔ STS−GS (95% CI), p^†^
Standing and walking time (min/day)	84.2	93.7	9.4 [−2.7, 21.6], p = 0.126	81.5	82.2	0.7 [−11.8, 13.2], p = 0.911	8.8 [−8.7, 26.2], p = 0.326
Sitting and lying time (min/day)	1357.0	1347.1	−10.9 [−24.4, 2.6], p = 0.115	1358.9	1357.1	−1.8 [−15.9, 12.4], p = 0.808	−9.1 [−28.7, 10.4], p = 0.361
Number of sit-to-stand transitions (n/day)	31.7	38.4	6.8 [1.5, 12.1], p = 0.013	33.7	37.3	3.6 [−1.5, 8.8], p = 0.167	3.1 [−4.3, 10.6], p = 0.408
Number of sitting bouts ≥30 min (n/day)	8.4	7.0	−1.4 [−3.1, 0.4], p = 0.125	8.1	9.3	1.2 [−0.7, 3.0], p = 0.209	−2.5 [−5.0, −0.1], p = 0.049
Total duration of sitting bouts ≥30 min (min/day)	710.3	581.2	−129.1 [−263.1, 4.8], p = 0.059	710.8	721.5	10.7 [−128.5, 149.9], p = 0.880	−139.9 [−333.2, 53.5], p = 0.156
Number of sitting bouts ≥60 min (n/day)	4.5	3.9	−0.6 [−2.1, 0.9], p = 0.446	4.8	4.4	−0.3 [−1.4, 0.7], p = 0.553	−0.3 [−2.0, 1.4], p = 0.752
Total duration of sitting bouts ≥60 min (min/day)	546.7	450.0	−96.6 [−228.6, 35.3], p = 0.151	552.0	497.8	−54.1 [−191.3, 83.0], p = 0.439	−42.5 [−232.9, 147.9], p = 0.662

Estimated marginal means (EMMs) at pre- and post-intervention are shown for each intervention (STS and goal-setting). Within-intervention changes (Δ, post − pre) and the between-intervention difference in change (ΔΔ, STS − goal-setting) were derived from linear mixed-effects models including sequence, period, intervention, time, and the intervention × time interaction as fixed effects, with participants as random intercepts.

^†^The p-value for ΔΔ corresponds to the formal test of the intervention × time interaction in the mixed-effects model.

Model-based estimates are presented. Raw observed means (±SD) for each assessment (Period 1 and Period 2; Pre and Post) are provided in Supplementary Table S1.

STS, sit-to-stand exercise; EMM, Estimated marginal means; GS, goal-setting; CI, confidence interval; SD, standard deviation

Model-based estimates are presented in Table 2; raw observed means (±standard deviation [SD]) for all 4 assessments (Period 1 and Period 2; Pre and Post) are provided in Supplementary Table S1.

### Standing/walking and sitting/lying time

There were no significant intervention, time, or interaction effects for total standing/walking time or total sitting/lying time (all p >0.05).

### Sit-to-stand transitions

Sit-to-stand transitions increased significantly during the STS instruction phase (31.7–38.4/day; Δ = 6.8, 95% CI [1.5, 12.1], p = 0.013), whereas the increase during the goal-setting phase was not statistically significant (33.7– 37.3/day; Δ = 3.6, 95% CI [−1.5, 8.8], p = 0.167). The intervention × time interaction was not significant (ΔΔ = 3.1, 95% CI [−4.3, 10.6], p = 0.408).

### Prolonged sitting bouts ≥30 min

A significant intervention × time interaction was observed for the number of prolonged sitting bouts ≥30 min (p = 0.049), suggesting that the pre–post change differed between the interventions (STS: 8.4–7.0/day; goal-setting: 8.1–9.3/day). The intervention × time interaction for the total duration of ≥30-min bouts was not statistically significant (p = 0.156).

### Prolonged sitting bouts ≥60 min

No significant intervention × time interactions were observed for the number or total duration of ≥60-min sitting bouts (all p >0.05). Both interventions showed small reductions from pre to post, with numerically greater decreases during the STS phase (−0.6 bouts/day and −96.6 min/day) compared with the goal-setting phase (−0.3 bouts/day and −54.1 min/day); however, these between-intervention differences were not statistically significant.

## Discussion

This study examined whether STS instruction or participant-led goal-setting could modify sedentary behavior patterns in chronic stroke survivors with reduced walking ability. A significant intervention × time interaction indicated that the change in prolonged sitting bouts ≥30 min differed between interventions. Total sedentary time and standing/walking time were unchanged, suggesting that sedentary pattern modification may occur even when overall activity levels remain stable.

Interrupting sedentary time may be a more feasible behavioral target than increasing ambulatory activity for individuals with substantial mobility limitations. Walking speed strongly influences step count^9)^, and slow walkers typically accumulate high sedentary time^10)^. Practicing sit-to-stand movements directly increases postural transitions, offering an immediate means of interrupting sitting. This is consistent with feasibility studies showing that standing- or transition-based approaches can be implemented safely^13,14)^. In contrast, participant-led goal-setting relies on autonomous behavioral regulation, which may yield less immediate change in individuals with impaired mobility or reduced confidence in self-directed behavior^17–19)^. These mechanistic differences likely contributed to the contrasting intervention effects.

The present findings are similar to prior observations of high sedentary time in stroke survivors^3–6)^ and add evidence that sedentary patterns—not overall sedentary volume—may be more modifiable in chronic survivors with reduced gait ability. Previous sedentary behavior interventions have shown mixed outcomes in chronic stroke^12,15,16)^, and few studies have directly compared a task-oriented motor strategy with a behavioral strategy. Our results suggest that a low-burden motor task may have greater potential to alter sedentary patterns than self-directed behavioral planning in this population, although superiority was not demonstrated.

Clinically, STS instruction is simple, low-cost, and home-based, making it feasible for routine use. Although total sedentary time did not change, reducing prolonged sitting may still yield benefits, as greater sedentary fragmentation is associated with better physical function^24)^ and sedentary behavior contributes to reduced muscle strength and power^25)^. Brief, achievable movements to interrupt sitting may help mitigate sedentary risks when meaningful increases in physical activity are unrealistic.

This study has limitations. The sample size was small, and carryover effects cannot be excluded despite the 1-month washout. Because sleep periods were included in the analysis, this may have introduced variability. The intervention duration was short, whereas habit formation often requires weeks to months^26)^. Goal-setting was intentionally delivered without feedback to allow a strict comparison with the motor task; more intensive support may produce different results.

## Conclusions

Sit-to-stand exercise instruction may contribute to modifying prolonged sitting patterns in chronic stroke survivors with limited walking ability, despite no significant changes in total sedentary or upright time. These preliminary findings suggest that a simple, task-oriented approach may help modify sedentary behavior patterns, although clear superiority over participant-led goal-setting was not demonstrated. Longer and larger trials are warranted.

## Supplementary Material

Supplementary Table S1. Observed means (±SD) for each assessment (Period 1 and Period 2; Pre and Post) according to intervention.
