# Supplementary material for: Effects of Sit-to-stand Exercise Instruction and Goal-setting on Reducing Sitting Time in Chronic Stroke Survivors: A Randomized Crossover Trial
Source: Phys Ther Res. 2026 Jun 10;29(2):143–9. doi: 10.1298/ptr.25-E10362 (PMC13624552; doi:10.1298/ptr.25-E10362)
Supplement: Supplementary file 1 [file ptr-29-143-s001.pdf]

Supplementary Table S1. Observed means (±SD) for each assessment (Period 1 and Period 2; Pre and Post) according to intervention

Sequence A: STS → GS (Period 1 = STS, Period 2 = GS)
Sequence B: GS → STS (Period 1 = GS, Period 2 = STS)
Values are mean ± SD

Outcome	Sequence	Period (Intervention)	Pre	Post
Standing and walking time (min/day)	A	Period 1 (STS)	108.5 ± 85.2	124.1 ± 101.2
Standing and walking time (min/day)	A	Period 2 (GS)	97.5 ± 109.4	110.8 ± 106.6
Standing and walking time (min/day)	B	Period 1 (GS)	65.2 ± 39.2	61.8 ± 47.4
Standing and walking time (min/day)	B	Period 2 (STS)	59.0 ± 41.1	66.3 ± 38.6
Sitting and lying time (min/day)	A	Period 1 (STS)	1333.4 ± 110.4	1326.6 ± 103.4
Sitting and lying time (min/day)	A	Period 2 (GS)	1342.8 ± 122.4	1329.7 ± 114.8
Sitting and lying time (min/day)	B	Period 1 (GS)	1377.3 ± 46.9	1361.8 ± 51.3
Sitting and lying time (min/day)	B	Period 2 (STS)	1375.0 ± 52.0	1371.6 ± 51.0
Number of sit-to-stand transitions (n/day)	A	Period 1 (STS)	25.2 ± 9.4	32.2 ± 12.5
Number of sit-to-stand transitions (n/day)	A	Period 2 (GS)	27.7 ± 11.8	33.1 ± 24.1
Number of sit-to-stand transitions (n/day)	B	Period 1 (GS)	39.8 ± 12.0	42.8 ± 10.9
Number of sit-to-stand transitions (n/day)	B	Period 2 (STS)	36.2 ± 14.2	43.4 ± 10.8
Number of sitting bouts ≥30 min (n/day)	A	Period 1 (STS)	6.2 ± 3.0	6.4 ± 1.4
Number of sitting bouts ≥30 min (n/day)	A	Period 2 (GS)	9.0 ± 4.5	9.7 ± 3.2
Number of sitting bouts ≥30 min (n/day)	B	Period 1 (GS)	8.6 ± 1.9	9.9 ± 3.0
Number of sitting bouts ≥30 min (n/day)	B	Period 2 (STS)	10.8 ± 2.3	8.3 ± 4.9
Total duration of sitting bouts ≥30 min (min/day)	A	Period 1 (STS)	470.6 ± 210.2	492.4 ± 183.0
Total duration of sitting bouts ≥30 min (min/day)	A	Period 2 (GS)	575.3 ± 289.3	664.5 ± 218.0
Total duration of sitting bouts ≥30 min (min/day)	B	Period 1 (GS)	846.6 ± 268.1	840.9 ± 298.0
Total duration of sitting bouts ≥30 min (min/day)	B	Period 2 (STS)	918.7 ± 237.4	770.0 ± 344.4
Number of sitting bouts ≥60 min (n/day)	A	Period 1 (STS)	2.6 ± 1.4	3.2 ± 1.2
Number of sitting bouts ≥60 min (n/day)	A	Period 2 (GS)	3.3 ± 2.2	3.6 ± 1.9
Number of sitting bouts ≥60 min (n/day)	B	Period 1 (GS)	5.4 ± 1.3	5.0 ± 1.8
Number of sitting bouts ≥60 min (n/day)	B	Period 2 (STS)	6.3 ± 2.2	5.6 ± 4.1
Total duration of sitting bouts ≥60 min (min/day)	A	Period 1 (STS)	317.3 ± 242.6	329.4 ± 210.2
Total duration of sitting bouts ≥60 min (min/day)	A	Period 2 (GS)	376.9 ± 270.5	392.5 ± 283.1
Total duration of sitting bouts ≥60 min (min/day)	B	Period 1 (GS)	662.3 ± 252.4	646.1 ± 298.0
Total duration of sitting bouts ≥60 min (min/day)	B	Period 2 (STS)	725.5 ± 319.0	548.6 ± 520.9

STS, sit-to-stand exercise instruction; GS, goal-setting; SD, standard deviation.
